# Long-term outcome of radiological-guided insertion of implanted central venous access port devices (CVAPD) for the delivery of chemotherapy in cancer patients: institutional experience and review of the literature

**DOI:** 10.1038/sj.bjc.6602082

**Published:** 2004-08-17

**Authors:** J Vardy, K Engelhardt, K Cox, J Jacquet, A McDade, M Boyer, P Beale, M Stockler, R Loneragan, B Dennien, R Waugh, S J Clarke

**Affiliations:** 1 Royal Prince Alfred Hospital, Missenden Rd, Camperdown, 2050 NSW, Australia; 2 Concord Repatriation General Hospital, Hospital Rd, Concord, 2139 NSW, Australia

**Keywords:** central venous access port devices, interventional radiologists

## Abstract

Central venous access port devices (CVAPD) are necessary for delivery of prolonged infusional chemotherapy or in patients with poor peripheral venous access. Previous studies of Hickman catheters report complication rates in about 45% of patients. Our aim was to assess the early and late complication rate, and duration that the CVAPD remained functional, following insertion by interventional radiologists in patients with solid tumours. A prospective study was undertaken in 110 consecutive patients who had insertion of 111 subclavian CVAPD. The median age of patients was 57 years (range 17–83), 64 were females; 68 patients (61%) had gastrointestinal tumours and 25 (23%) had breast cancer. CVAPD were successfully implanted in all but one patient. There were four (4%) immediate major complications: thrombosis 2 and pneumothorax 2. Nine patients (8%) had bruising or pain. Four devices (4%) became infected. In total, 100 CVAPD (90%) were either removed as planned at the end of treatment (*n*=23) after a median 203 days, or remained *in situ* for a median of 237 days (7–1133). Premature removal occurred in eight patients due to infection (*n*=4), thrombosis (*n*=3) or faulty device (*n*=1). Four patients were lost to follow-up. Radiological insertion of CVAPD is safe and convenient with low rates of complications.

Central venous access devices have been used to deliver chemotherapy since the 1970s. In patients with solid cancers they are used mainly to overcome poor peripheral venous access or for prolonged infusional chemotherapy.

There are three types of central venous devices: external central catheters (e.g. Hickman's catheters), peripherally inserted central catheters (PICC) and subcutaneously implanted venous access devices. External catheters are desirable when multiple lumens are necessary for the concurrent administration of different solutions, but they are associated with high rates of catheter-related infection and thrombosis; reported complication rates are as high as 45% (Eastridge and Lefor, 1995; Biffi *et al*, 1997, 1998). Patients with external catheters also require more frequent catheter irrigation and dressing changes than those with fully implanted venous access devices. While the initial cost of CVAPD is high (Biffi *et al*, 1998), a case–control study comparing durability and cost of CVAPD and external catheters demonstrated long-term economic benefit for CVAPD for use beyond 6 months due to lower ongoing maintenance costs (McCready *et al*, 1991). Peripheral external catheters are relatively inexpensive and can be inserted by trained nurses at the bedside, however, they are only a short-term option, with similar complications to Hickman catheters.

Implanted venous access devices can be inserted either peripherally near the antecubital fossa (Pasports) or centrally into the subclavian or jugular vein (CVAPD). Instead of an external catheter the tunneled catheter terminates into a subcutaneously implanted reservoir. This makes them suitable for active patients and more acceptable cosmetically (Lokich *et al*, 1985; Biffi *et al*, 1998; Lyon *et al*, 1999). Peripheral ports have a lower risk of infection than CVAPD (Salem *et al*, 1993; Damascelli *et al*, 1997) and their insertion involves a minimal risk of pneumothorax and haemothorax. However, they have a shorter useful lifetime than CVAPD (Smith *et al*, 1998) and there is an increased risk of venous sclerosis following the use of cytotoxic agents (Slater *et al*, 1985), which makes them unsuitable for cancer patients receiving longer-term chemotherapy.

Traditionally, CVAPD have been implanted by surgeons or anaesthetists in an operating room. More recently, interventional radiologists have been inserting them using local anaesthesia and fluoroscopic guidance. This method is more efficient, and recent studies including our own, have demonstrated a favourable success rate and low complication rate following radiological insertion of portacaths (Morris *et al*, 1992; Simpson *et al*, 1997; Lorch *et al*, 2001).

The data presented here represents the initial experience with 110 cancer patients undergoing outpatient insertion of CVAPD by radiologists, for the administration of chemotherapy, at two Sydney teaching hospitals. The present study differs from published studies of long-term indwelling catheters (reviewed below) in that it is prospective and so avoids the numerous attendant problems associated with retrospective collection of data. Our study population is relatively homogenous consisting of patients with solid tumours, who require long-term indwelling venous access for chemotherapy, in contrast to other studies which include patients with solid and haematological malignancies, and/or those with HIV or chronic infections requiring long-term antibiotics. Oncology patients are at higher risk than the general medical population for complications such as thrombosis; but at lower risk than acute leukaemic patients for sepsis. In addition, our study evaluates only CVAPD inserted as an outpatient procedure by interventional radiologists. A number of the other studies include a mixture of different types of venous access devices, so that comparison of complication rates is difficult. Finally, our study is not only prospective but also has a long follow-up (minimum of 18 months).

## MATERIALS AND METHODS

### Patient selection

All patients had histologically confirmed malignancy other than leukaemia and required the insertion of a CVAPD for the administration of chemotherapy. Approval for the study was obtained from the Institutional Review Board, and all patients gave informed consent.

The CVAPD were inserted with fluoroscopic guidance by one of three interventional radiologists as an outpatient procedure. All CVAPDs were inserted into the subclavian vein. When possible, the right subclavian vein was used for technical ease of right-handed operators. In all, 54 patients had insertion of a BardPort® with an 8F Groshong® catheter (Bard Inc., Salt Lake City, USA), while 57 had a Vital-Port® with a 6.5F silicone catheter (Cook Vascular Corporation, Pennsylvania, USA). A full blood count and coagulation studies were performed prior to the procedure and patients and their carers received education prior to and following CVAPD insertion.

At the commencement of the procedure, patients received midazolam 2 mg i.v. and pethidine 25–100 mg i.v., with further increments of midazolam being administered as required during the procedure. Local anaesthetic was injected into the skin surrounding the planned puncture site after preparation of the skin. All patients received cephalothin 1 g i.v. as prophylaxis against infection or 300 mg oral clindamycin if there was a history of penicillin/cephalosporin allergy. A mixture of 50 ml of contrast (Ultravist 370) and saline 30 ml was injected peripherally to identify the site of subclavian vein puncture. A postprocedural chest X-ray (CXR) was performed to document final catheter position and to exclude pneumothorax. Warfarin 1 mg orally daily was commenced after CVAPD insertion in order to prevent catheter thrombosis (Bern *et al*, 1990).

The patients and CVAPD were assessed by Oncology nursing staff immediately postinsertion and most CVAPDs were accessed immediately. Follow-up assessment occurred at intervals of 4 weeks or less. The patient was questioned about symptoms suggestive of infection and problems with catheter function, and the insertion site was examined for evidence of infection, bruising or other complication. Patients were followed until the device was removed or the patient died. Complications were classified as acute if they occurred within 1 week of insertion of the device, and delayed if they occurred after this. CVAPD infection was defined as a positive blood culture with no other obvious source of infection.

### Statistical methods

Descriptive statistics were used to summarise demographic and CVAPD characteristics. Analysis involved the use of simple summary statistics. Actuarial curves describing retention of functional CVAPD was estimated using the Kaplan–Meier method. A successful CVAPD was one that was still *in situ* and working, at the time of death of a patient or removal of a functional port, as planned upon completion of chemotherapy treatment.

## RESULTS

In all, 111 CVAPD were inserted in 110 patients from June 1998 to July 2001. There were 46 males and 64 females ranging from 17–83 years old with a median age of 57 years. The predominant malignancies were gastrointestinal (61%) and breast cancer (23%). Overall, 99 CVAPD were inserted on the right side and 12 on the left.

Complications associated with the CVAPD are summarised in Table 1

**Table 1:** Summary of CVAPD complications

	**%**
*Acute complications*	
Major	
Inability to insert	1
Thrombosis	2
Pneumothorax requiring ICC^a^	2
Minor	
Haematoma	4.5
Pain at port site	4.5
	
*Delayed complications*	
Infection → port removal	4
Infection → antibiotics	2
Thrombosis	3

a ICC= intercostal catheter.

. CVAPD were successfully implanted in all but one patient (1%) where there was device failure with the catheter disengaging from the port, necessitating immediate removal in the operating room. Most CVAPD (*n*=108, 97%) were inserted with a single pass. Acutely there were four major complications (4%). Two patients (2%) developed thromboses, which were successfully treated with anticoagulation and did not require port removal, and two (2%) patients developed a pneumothorax requiring insertion of an intercostal catheter. Totally, 10 patients (9%) had minor acute complications: five (4.5%) developed a haematoma at the site of the port insertion, and five (4.5%) described moderate–severe pain at the port site, requiring analgesia.

Delayed complications requiring removal of the port occurred in seven patients (6.3%). Four patients (4%) developed infections necessitating removal of their CVAPD: three secondary to *Staphylococcus aureus* infection (days 12, 21 and 282) and one *Pseudomonas aeruginosa* requiring port removal at day 611. Two (2%) additional patients had *S. aureus* infection successfully treated with antibiotics. Two patients (2%) developed delayed thrombosis requiring removal of the port (days 61 and 127); one of them had prior thrombosis treated with anticoagulants. The other patient had port removal and a second port inserted immediately.

**Figure 1:**
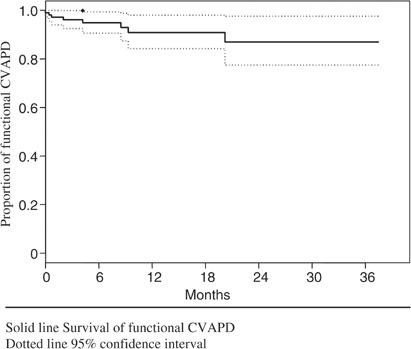
Actuarial curves describing retention of functional CVAPD. Solid line=survival of functional CVAPD. Dotted line=95% confidence interval.

In total, 100 (90%) CVAPD served their intended purpose for as long as they were required. In all, 26 (23%) functional CVAPD were removed as planned upon completion of chemotherapy, with a median duration of insertion of 7 months (range 2–19 months). Overall, 63 (60%) patients died with a functional CVAPD *in situ* at a median of 7 months (range 0–33 months), and 11 (10%) patients were alive with a functional CVAPD *in situ* at the conclusion of the study (median duration of insertion=30 months; range 25–37). Thus, 74 (67%) CVAPD remained *in situ* for a median of 8 months (range 0–37 months). Four (3.6%) patients were lost to follow-up. At 2 years there is a one in six chance that a CVAPD will need to be removed (see Figure 1

).

## DISCUSSION

CVAPD are used increasingly in the treatment of cancer patients for administration of continuous infusional chemotherapy; and to avoid frequent venipunctures, which become increasingly difficult after multiple courses of chemotherapy. Patients prefer the cosmetic result of a fully implanted venous device compared to a central venous device with external lines (Lyon *et al*, 1999). The implanted device is particularly desirable for patients with active lifestyles and they require less maintenance than external tunneled catheters. A major advantage of having the CVAPD inserted by an interventional radiologist is that it is an outpatient procedure without need for operating room time.

Table 2

**Table 2:** Summary of venous access device studies

**Author**	**Year**	**No of devices**	**Inserted by**	**Type of device**	**Infection (%)**	**Thrombosis (%)**	**Pneumothorax (%)**	**Device malfunction (%)**	**Removal for Complication(%)**	**Mean catheter life (months)**	**Months of service**
Vardy,	2004	111	Radiology	CVAPD	4	2	2	1	7	7	1222
Lorch *et al*,	2001	125	Radiology	CVAPD	2.4	0	1.6	2.4	4.8	3	364
de Gregorio *et al*,	1996	288	Radiology	CVAPD	4.1	4.5	0.7	1.4	18	10	2984
Biffi *et al*,	1998	333	Surgery	CVAPD	2.7	1.5	3.4	2.7	3.6	8	2605
Schwarz *et al*,	1997	680	Surgery	CVAPD	8.8	3	0	1.7	14.1	39	N/A
Kock *et al*,	1998	1500	Surgery	CVAPD	3.2	2.5	0.3	2.8	11.9	9	14013
Eastridge and Lefor,	1995	113	Surgery	CVAPD	5	6	1.2 total	6% total	13.2	N/A	N/A
		209	Surgery	Catheters	13	10			32	N/A	N/A
Damascelli *et al*,	1997	30	Radiology	CVAPD	8.3	0	Total 3%	2.8	5.5	8	809
		111	Radiology	Catheters	6.3	1.8		1.8	10.8		
Groeger *et al*,	1993	788	Surgery	Catheters	43	N/A	N/A	N/A	N/A	N/A	5451
		680	Surgery	CVAPD	8	N/A	N/A	N/A	N/A	N/A	9140
Gertner *et al*,	2000	278	N/A	Catheters	12.5	1.5	N/A	5.2	N/A	5	N/A
		110	N/A	CVAPD	4.6	1.1	N/A	0	N/A	7	N/A
Tolar and Gould,	1996	324	Surgery	Catheters	19.4	16.6	N/A	33.4	N/A	6	1969
Broadwater *et al*,	1990	763	Surgery	Catheters	4	3	2	N/A	25	6	N/A
Nightingale *et al*,	1997	949	Surgery	Catheters	10.6	4.4	1.8	2.2	18.6	4	3273
Smith *et al*,	1998	283	Surgery	Catheters and CVAPD	11.7	0.7	1.4	3.9	N/A	7	1624
		555	Nurses	PICCs	8.1	2.5	0	11.7	N/A	23	389
Walshe *et al*,	2002	351	Nurses/Rad	PICCS	7.4	3.4	0	6	32.8	1	347
Marcy *et al*,	2001	652	Radiology	Peripheral ports	0.9	1.2	0	1.8	4	5	N/A
Lyon *et al*,	1999	195	Radiology	Peripheral ports	5.1	4.6	0	5.1	9.7	6	1105
Hills *et al*,	1997	100	Radiology	Peripheral ports	7	0	0	0	6.2	8	784

N/A=not available.

summarises the complication rates of the larger published studies of CVAPD inserted by radiologists and surgeons, as well as studies of external catheters and peripheral ports. There is little standardisation of the definitions used for complications. For example, some studies restrict the definition of infection to those patients with proven positive cultures, while others include patients with fever and/or clinical evidence of infection despite negative cultures. A number of the studies only included a complication if it led to major sequelae such as the venous access device being removed. An attempt has been made to address this problem with the development of Reporting Standards for Central Venous Access (Silberzweig *et al*, 2000).

In our study, 4% of patients developed immediate complications of thrombosis or pneumothorax. Our rates are consistent with other published studies of CVAPD inserted by interventional radiologists. The range of complication rates for CVAPD inserted by surgeons is generally higher (Table 2). Thus, the method of insertion may affect the outcome.

A number of the studies attempted to compare CVAPD with either Groshong catheters or Hickman catheters and consistently found a higher rate of complications in the central external catheters (Mueller *et al*, 1992; Gleeson *et al*, 1993; Groeger *et al*, 1993; Eastridge and Lefor, 1995) (Table 2).

Complication rates of peripheral catheter (PICCS) are shown in Table 2. Walshe's study report 6.6% of PICCS removed for phlebitis. Complication rates of peripheral passports are similar to CVAPD with the exception of lower rates for pneumothorax (Table 2).

Direct comparisons between the studies summarised in Table 2 are difficult, because they were applied to different patient populations with different disease states. There is an increased rate of thrombosis in cancer patients and complication rates in cancer patients are likely to depend on the type of malignancy and its attendant treatment. Patients with cancer having lines inserted for administration of chemotherapy will commonly become neutropenic leading to a higher risk of line infection than in general medical patients (Walshe *et al*, 2002).

Despite the above difficulties, the consensus of the studies reviewed in Table 2 supports the use of CVAPD over external catheters for patients. The low complication rate associated with CVAPD ensures that a large majority of patients is able to complete their chemotherapy without delays.

Our experience indicates that insertion of CVAPD by interventional radiologists has resulted in less time delays for patients commencing treatment than we used to experience with surgical insertion. This is due primarily to not having to wait for operating room time.

Our prospective study of solid tumour patients has found radiological insertion of CVAPD to be safe and to compare favourably with surgical insertion. Insertion by interventional radiologists has been more convenient and is favoured by both medical oncologists and patients. With the increased use of continuous infusional chemotherapy regimes there is likely to be a substantial increase in the requirement for CVAPD.
